# Histological, Histomorphometrical, and Biomechanical Studies of Bone-Implanted Medical Devices: Hard Resin Embedding

**DOI:** 10.1155/2020/1804630

**Published:** 2020-01-17

**Authors:** M. Maglio, F. Salamanna, S. Brogini, V. Borsari, S. Pagani, N. Nicoli Aldini, G. Giavaresi, M. Fini

**Affiliations:** ^1^IRCCS Istituto Ortopedico Rizzoli, Laboratory of Preclinical and Surgical Studies, Bologna, Italy; ^2^IRCCS Istituto Ortopedico Rizzoli, Laboratory of Biomechanics and Technology Innovation, Bologna, Italy

## Abstract

The growing incidence of degenerative musculoskeletal disorders as well as lifestyle changes has led to an increase in the surgical procedures involving implanted medical devices in orthopedics. When studying implant/tissue interface in hard materials (i.e., metals or dense plastics) and/or in large bone segments, the hard plastic embedding of the intact undecalcified tissue envelope with the implant *in situ* is needed. The aim of this work is to describe the advances and the possibilities of high-temperature methyl methacrylate (MMA) embedding for the histological, histomorphometrical, and biomechanical assessment of bone-implanted medical devices. Unlike routine techniques, undecalcified bone processing histology, using high-temperature MMA, requires a complex and precise sample processing methodology and the availability of sophisticated equipment and software for both sample preparation and analyses. MMA embedding permits the evaluation of biological responses to the presence of implanted medical devices without implant removal, allowing simultaneous qualitative and quantitative histological evaluation, both static and dynamic histomorphometry, and biomechanical analyses not possible with tissue decalcification. MMA embedding, despite being a demanding procedure, is still preferred to other kinds of resin-based embedding because of its peculiar characteristics, which allow the study of samples of big dimensions also implanted with hard materials without reducing the sample or removing the material. Dynamic measurements are allowed together with biomechanical investigations at the bone-biomaterial interface, obtaining a comprehensive and precise evaluation of the safety and effectiveness of medical devices for orthopedic regenerative, reconstructive, and reparative surgery.

## 1. Introduction

Orthopedic medical devices have been extremely successful in restoring mobility, reducing pain, and improving the quality of life of millions of individuals each year [1, 2]. Nowadays, different kinds of synthetic or composite materials with complex topographical features and manufacturing processing techniques are developed as bone implants and scaffolds for regenerative, reconstructive, and reparative medicine [3]. The final step of the medical device approval for the patient's use goes through an exhaustive regulatory process to prevent clinical complications or any other close or distant side effects. In this regulatory process, testing a novel material involves *in vitro* assays with cell cultures and *in vivo* tests using proper animal models. The biocompatibility, i.e., the process of evaluating materials used in the manufacture of medical devices (or a material component of such), is guided by a series of Standards International ISO 10993.

In biomedical research, when *in vitro* studies are inapplicable or not exhaustive, animal studies are mandatory to investigate the safety and to establish proof of burden on the feasibility and preclinical efficacy of medical devices as biomaterials, scaffolds, bone substitutes, and engineered constructs. In general, small laboratory animals are preferred due to the greater simplicity of management and housing and for precisely answering biological mechanisms. The use of large animals may be justified based on special scientific considerations of the particular material under study, or if needed to accommodate implant size, with whole device testing, or to satisfy particular pathophysiological and loading conditions.

When implant/tissue interface of hard materials (i.e., metals, dense plastics) is to be studied and/or if large animals have been used with the implantation of scaled-up materials, the embedding of the intact tissue envelope with the implant *in situ* using hard plastic is needed. Plastic embedding for undecalcified bone tissues is a well-established strategy for histopathological and histomorphometric study of samples which cannot be properly evaluated with classical paraffin embedding of decalcified bone tissues. To date, high-temperature methyl methacrylate (MMA) embedding, despite being a demanding procedure, is still preferred to other kinds of resin-based embedding due to its peculiar characteristics. When compared, for example, to water-soluble MMA, such as glycol methacrylate (GMA), high-temperature MMA proves to better infiltrate samples of big dimensions and at the same time can be removed by sections in order to improve staining quality and the evaluation of tissue morphology [4]. Also, in comparison with low-temperature MMA-based resins, which allows us to obtain thinner sections and therefore a better histopathological evaluation of the section, the inclusion in high-temperature MMA could be preferred. In fact, it allows us to evaluate samples of much larger sizes in relatively shorter times and with less labor-intensive passages, since other resins allow the evaluation of samples up to 1.5 cm × 1.5 cm. However, the adoption of high-temperature MMA-based embedding solutions for histology requires that laboratories set up appropriate working spaces for all processing and embedding steps and for the subsequent phases of realization of the histological slides. The presence of adequate chemical fume hoods is mandatory for the safe manipulation and preparation, not only for formaldehyde-based fixatives but also for MMA-based infiltrating and embedding solutions, so as to avoid or minimize the exposure to vapors of monomer. In addition, the disposal of the MMA monomer and the first infiltration solution requires specific procedures. In comparison to other most common embedding techniques, such as paraffin, high-temperature MMA embedding foresees longer and alcohol consuming steps for dehydration and requires further reagents for embedding steps. Considering the high temperature of MMA polymerization, special embedding cassettes/molds resistant to temperature are needed for sample inclusion and much more attention must be paid to sample orientation because, differently from wax, MMA polymerization is an irreversible reaction. The quality of the final MMA-embedded sample depends on the correct performance of all the above-mentioned procedures; only after perfect processing, a variety of histological, histomorphometric, and biomechanical evaluations can be carried out.

Since histology, histomorphometry, and biomechanics are key methodologies for bone-implanted medical device evaluation, it is extremely important to update the concept of “hard resin embedding” as a method not allowing precise evaluations. The purpose of this work is to outline a method for the preparation of bone-implanted medical devices embedded in high-temperature MMA and to highlight the evaluations that this technique allows to carry out.

## 2. Materials and Methods

Once the bone-implanted medical device is collected, it is crucial to promptly carry out the processing steps in order to prevent the deterioration of the tissue triggered by proteolytic enzymes and, at a later stage, to preserve its microarchitecture, making it more resistant to subsequent processing phases.

### 2.1. Fixation and Dehydration

Fixation is used to prevent tissue decomposition and preservation of cell and matrix structure and to intensify subsequent staining. Bone-implanted medical devices must be fixed immediately after harvesting to avoid artifacts. Although many fixatives are available, these samples are usually fixed in 4% paraformaldehyde at room temperature. As it is essential to put samples in an excess of fixative, starting from a minimum acceptable fixative/tissue ratio of 1 : 20, also fixation times depend on sample dimensions. The minimum fixation time is considered 24 hours. After the fixation period, bone-implanted medical devices are extensively rinsed in distilled water, for at least 4–6 hours, with frequent changing of water, and then washed in running tap water for another 4 hours to eliminate all the fixative. To eliminate the entire water content of the sample, they are dehydrated in increasing concentration of alcohols in steps of at least 24 hours each, starting from 50% ethanol, 70%, twice 96%, and three times 100%, depending on sample dimension.

### 2.2. Embedding

After dehydration, the high-temperature MMA embedding procedure for bone-implanted medical devices is organized in the following basic steps:

1. Infiltration of the sample with MMA monomer for 24 hours
2. Infiltration of the sample with MMA with the addition of benzoyl peroxide and Tergitol in the solution for 24 hours
3. Inclusion in embedding solution: MMA with the addition of benzoyl peroxide and Tergitol, to which low molecular weight polymethylmethacrylate (PMMA) is slowly added

Once the complete suspension of the PMMA is achieved, taking into account the fact that the preparation of the final embedding solution can require some hours, samples are placed in the obtained solution within specific polyethylene cassettes/molds and finally oriented according to the cutting requirements. The polymerization process is an exothermic chain reaction and is carried out in strictly controlled temperatures, as the reaction can reach very high temperatures and be very violent, causing bubble formation inside the preparation and movement of the samples, risking altering the orientation predisposed for the following cutting phase. The polymerization time is also influenced by many factors besides temperatures, such as the sample dimension. Samples larger than 3 cm will take more time to complete embedding, also in the order of several weeks, while smaller samples can require just a few days. The whole polymerization phase, as well as MMA-based solution preparation and all infiltration steps, must be conducted under a chemical fume hood in order to avoid air dispersion of monomer when in liquid form.

### 2.3. Sectioning and Grinding

Once polymerized, the blocks are removed for their cassettes/molds and are sectioned by means of a surgical saw. Cutting systems are provided with diamond saw blades that can grind both fresh and embedded samples very precisely, with continuous water cooling, provided by a closed recirculation system, to avoid thermal changes that can get artifacts or friction burns. Allowing a perfect MMA “solidification” is mandatory for the successful execution of the subsequent steps; if MMA is still soft, cutting results are virtually impossible to be carried out with precision and the result in the dulling of the block surface, expected to be clear and diaphanous, can occur after contact with water. To reduce the cutting times and preserve the integrity of samples, the gripping system can oscillate and tool direction can be reverted, and a feed rate mechanism is present to adjust the cutting speed. These options are particularly useful in the presence of very hard implant like metallic materials, for which the slowing of the cutting speed and the oscillation of the sample facilitate the cutting phase, which is more difficult due to the hardness of the sample and the friction that is generated in contact with the blade. Sections obtained after cutting can usually have a thickness of 100 ± 10 *μ*m, which can be measured with a metric gauge for measuring thickness, subtracting the thickness of the histological slide. The obtained section must be pasted to plastic microscope slides of appropriate dimension with an adequate cyanoacrylate adhesive, with particular attention to avoid the formation of bubbles and to allow a perfect adhesion of sample to the slide in order to obtain a flat surface. This will make the subsequent steps of grinding and polishing easier and also have a clear field for the microscope observation, as well as achieving a better quality of histological preparation. Once adhesion is completed, after about 60 minutes, sections are thinned with an abrasion system using abrasive papers with different granulation, from 300 to 4000 grit (from more to less abrasive surface), in steps of about 15 minutes each, up to a thickness of 25 ± 10 *μ*m. This step is particularly challenging because excessive abrasion can cause the loosening of all or part of the sample, especially if the surface of the sample obtained after cutting is not completely equal or in the presence of metallic materials, which thin out more slowly than the surrounding bone. In addition, this procedure can cause the formation of lines on the surface of embedded samples, especially when the most abrasive papers are used for the thicker samples that have very hard implants. The sample surface is therefore smoothed by a polishing system using velvety cloths, sometimes in combination with diamond paste or alumina solution, indicated for surfaces difficult to polish and exposed to ceramic and ferrite materials [5–7].

### 2.4. Staining

**Figure 1:**
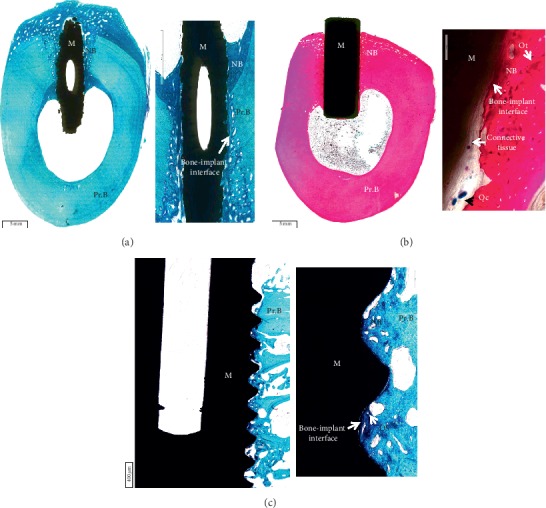
(a) Sheep tibial diaphysis six months after a roughened titanium alloy implantation. Toluidine Blue and Fast Green stain: in black, the material; in light green, the preexisting bone; in dark green, the newly formed bone. Left: magnification 1x; right: magnification 30x. (b) Sheep tibial diaphysis three months after a sandblasted titanium implantation. Stevenel's blue and picrofuchsin stain: in black, the material; in fuchsia, the bone; in blue, the cells. Left: magnification 1x; right: magnification 30x. (c) Sheep iliac crest three months after a titanium screw implantation. Toluidine Blue and Fast Green stain: in black, the material; in light green, the preexisting bone; in dark green, the newly formed bone. Left: magnification 10x; right: magnification 30x. M: material; NB: new bone; Pr.B: preexisting bone; Ot: osteocytes; Oc: osteoclasts; OS: osteoid tissue.

In general, staining methods for plastic-embedded tissue require modifications because these procedures are usually performed without the removal of the plastic, and thus different timing regimes are utilized. However, different staining protocols can be chosen for MMA-embedded bone and the choice can be influenced by the kind of evaluations to be performed. Staining procedures that are commonly used in our laboratory reflect the attempt to primary evaluate material osseointegration or osteoinduction. Both Von Kossa and Alizarin Red staining are usually preferred for the detection of calcium deposits. Goldner's trichrome, using also hematoxylin, can better identify cellular components and distinguish newly formed bone matrix (in red) from mature ones (in green) and calcified cartilage. Van Gieson staining allows us to detect the nuclei, colored in black, and to differentiate between osteoid and collagen (red) and bone and muscle in green and can be successfully applied for the evaluation of bone-implant interface, and Stevenel's blue staining allows us to assess the presence of fibrous tissue. Perhaps one of the most utilized stains remains Safranin O/Fast green, which allows us to stain cartilage in red and bone in green. The combination of Fast Green with Toluidine Blue allows us to detect nuclei in dark blue and to distinguish old bone from newly formed bone thanks to Fast Green intensity. In fact, the staining allows appreciating a chromatic difference between the preexistent bone, whose green coloration is milder, and the newly formed bone, which is more brilliant. Such a chromatic difference is well represented in Figure 1(a), in which the bone growth around and in contact with the metallic implant is stained more intensely than the preexisting bone.

## 3. Results

### 3.1. Histological Findings of Bone-Implanted Medical Devices

The quality of the final histological slide depends on the correct performance of all the procedures described above. Histological evaluation and characterization of bone-implanted medical devices are normally done with light microscopy. However, it is also possible to use a digital pathology slide scanner, a rapid and high-resolution scanner able to convert pathology glass slides into digital slides in few minutes, obtaining in a single acquisition an image that can be observed at different real optical magnifications. Being a digital technique with very stringent acquisition parameters, only partially modifiable by the operator, the recognition and correct focusing and acquisition of the sample are functions of the quality of the histological preparation, specifically in terms of slice thickness and effective staining.

The biological response parameters that can be assessed, also following the ISO 10993-6:2016-*Biological Evaluation of Medical Devices-Part 6*: *Tests for Local Effects after Implantation*, are numerous. The most used are changes in tissue morphology, presence of fibrosis and inflammatory cells, presence of necrosis, vascularization, fatty infiltration, foreign body reaction, mineralization, bone formation, maturation and density, materials fragmentation, bone quality, and bone ingrowth. In addition, the interface between the tissue and the materials is of critical interest. In fact, it is fundamental to evaluate the area of bone contact near the implant (osseointegration), as well as the presence of intervening noncalcified tissues. Obviously, the presence of bone resorption or new bone formation should be also recorded. Some examples of histological evaluation of bone-implanted medical devices are reported below.

**Figure 2:**
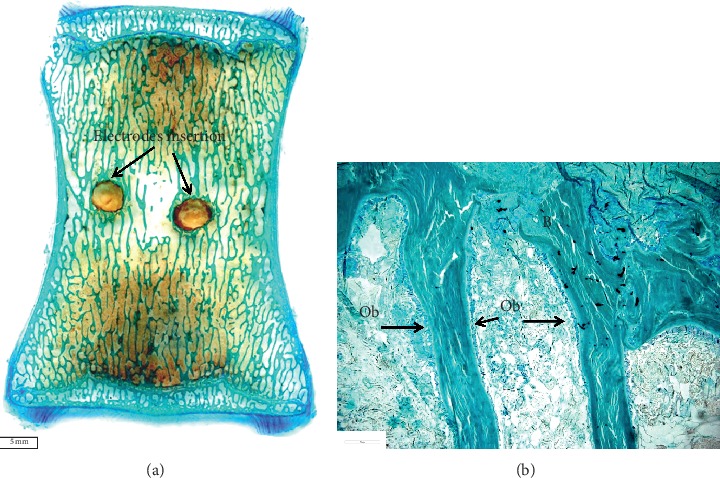
Sheep vertebra. Toluidine Blue and Fast Green stain. Ob: osteoblasts. (a) Magnification 1x; (b) magnification 30x.

**Figure 3:**
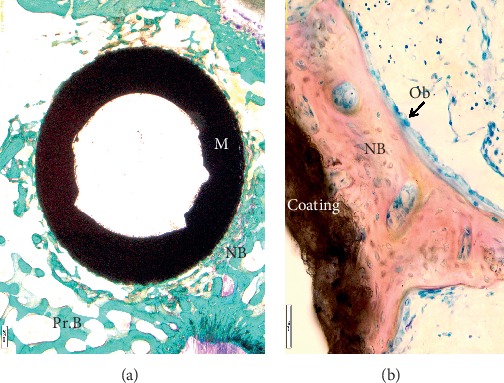
Hydroxyapatite-coated titanium implant in a rabbit femoral condyle. (a) Toluidine Blue and Fast Green stain: in black, the material; in green, the bone. Magnification 10x. (b) Stevenel's blue and picrofuchsin stain: in black, the material; in fuchsia, the bone; in blue, the cells. Magnification 40x. M: material; NB: new bone; Pr.B: preexisting bone; Ob: osteoblasts.

Figures 1(a) and 1(b) show the histological images of a roughened titanium alloy (Figure 1(a)) and of a sandblasted titanium implant (Figure 1(b)) implanted in a sheep tibial diaphysis. Microscopic analyses, at three and six months after implantation, showed that the gap between the preexisting cortical bone and the implant is filled with newly formed lamellar bone and direct bone-to-implant contact is observed for both implants. However, in the same area of Figure 1(b), when observed at higher magnification, the newly formed bone appeared separated from the implant by a thin layer of connective tissue. Osseointegration *per se* is not linked to any particular surface characteristics, because a great number of different surfaces achieve clinical osseointegration. However, stronger or weaker bone responses may be related to the surface characteristics. Nevertheless, it is important to emphasize that higher magnification images allowed us to detect no infection signs (polymorphonuclear cells, lymphocytes, macrophages, and multinucleated cells), no implant malposition, or implant loosening (Figure 1(b)). Another example is shown in Figure 1(c) that reports a histological image of a titanium screw implanted in a sheep femoral condyle. Histological analysis three months after implantation showed complete and good osseointegration of the implant and presence of newly formed trabecular bone and active osteoid tissue (noncalcified tissues) strictly associated with the implant surface. The new bone consisted primarily of woven bone, with the greatest density in the area closer to the native bone. The woven bone is often considered as a primitive type of bone tissue and characterized by random, felt-like orientation of its collagen fibrils, numerous, irregularly shaped osteocytes, and, at the beginning, a relatively low mineral density [8–10]. Another example is shown in Figure 2 where an entire sheep vertebra was histologically evaluated in order to assess the influence of the insertion procedure of two electrodes on bone tissue viability. At higher magnification, it is possible to see the presence of newly formed bone in the whole trabecular bone around the electrodes, thus highlighting that the insertion procedure does not influence bone tissue viability. In fact, preexisting trabeculae among the electrodes were covered by osteoblasts (OBs) with evident evenly spread osteocytes [11]. A hydroxyapatite-coated titanium implant (Figure 3) was implanted in a rabbit femoral condyle. The hydroxyapatite-coated titanium implant showed an excellent integration of the implant with newly formed trabecular bone covered by OBs and osteoid tissue (Figure 3).

**Figure 4:**
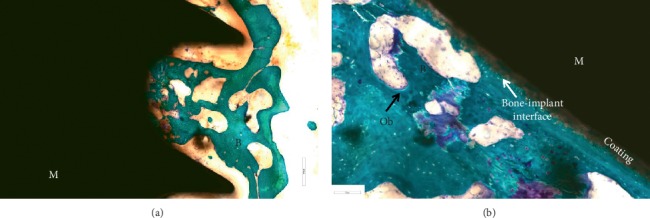
Human femoral neck hydroxyapatite-coated titanium screw. Toluidine Blue and Fast Green stain: in green, the bone; in black, the screw; in blue, the cells. M: material; B: bone; Ob: osteoblasts. (a) Magnification 1x. (b) Magnification 10x and 30x.

Another excellent example of MMA use is reported in Figure 4, a biological failure of a human femoral neck hydroxyapatite-coated titanium screw. Histological analysis of periprosthetic tissue reactions, including necrosis, lymphocyte infiltration, histiocytosis, and intracytoplasmic metallic debris, was carried out. In addition, the evaluation of the area of bone contact near the implant and the presence of intervening noncalcified tissues and the presence of bone resorption or new bone formation were also recorded. The histological evaluations of these kinds of devices allowed us to better understand the characteristics of bone quality and its microarchitecture even after their implantation. These aspects are of significant importance in the development and improvement of future medical devices.

### 3.2. Histomorphometric Findings of Bone-Implanted Medical Devices

**Figure 5:**
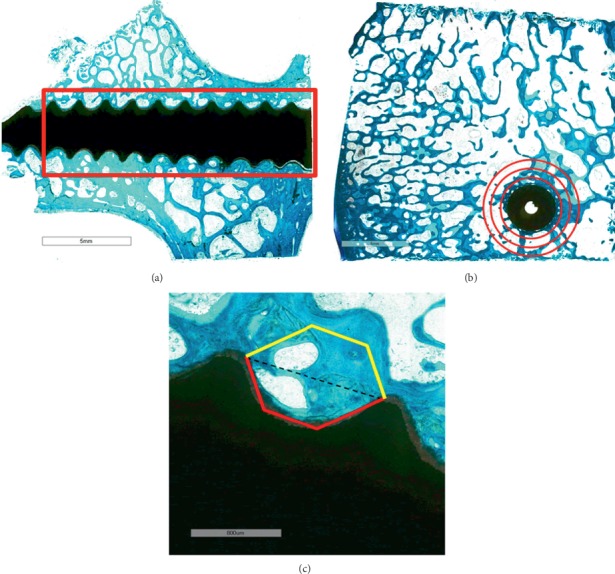
(a) Hydroxyapatite-coated titanium screw in sheep vertebral pedicle. The red rectangle indicates the region of interest for histomorphometric measures; Toluidine Blue/Fast Green staining; magnification 1x. (b) Cylindrical titanium implant in sagittal section in sheep iliac crest. The red circles identify regions of interest for histomorphometric measures; Toluidine Blue/Fast Green staining; magnification 1x. (c) Histomorphometrical measure of bone ingrowth and mirror area of hydroxyapatite-coated titanium screw implanted in sheep vertebral pedicle. In red, the ROI for the assessment of bone ingrowth between the two turns of the screw; in yellow, the ROI for the assessment of the mirror area; Toluidine Blue/Fast Green staining; magnification 10x.

**Figure 6:**
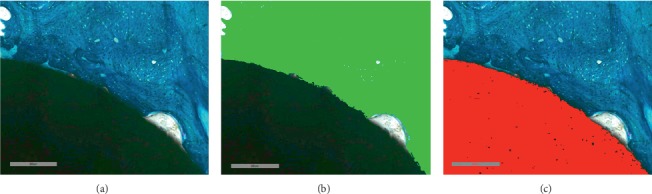
Image binarization of titanium cylindrical implant in sheep iliac crest. In sequence, (a) histological image of interface bone-implanted stained with Toluidine Blue/Fast Green; (b) binarization of bone tissue in green; (c) binarization of implant in red (magnification 20x).

**Figure 7:**
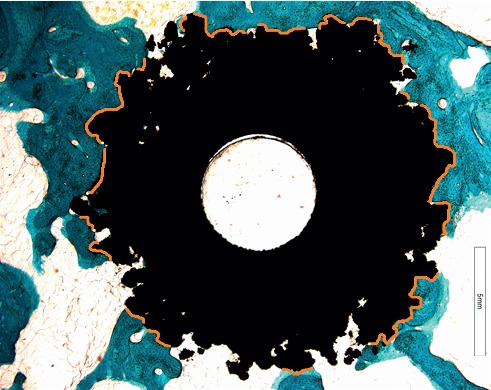
Evaluation of affinity index of a cylindrical titanium implant in sheep tibial epiphyses. The orange lines indicate the point of contact between the bone and the implanted material; Toluidine Blue/Fast Green staining; magnification 1x.

For the evaluation of bone-implanted medical devices, quantitative histomorphometry must be used in addition to the qualitative histological analysis that can suffer from operator-related bias and does not provide statistically assessable numerical data. Histomorphometry is defined as a methodology for quantitatively analyzing measurable histological parameters (as length, distance, area, number of components of interest, etc.). The first attempt to uniform the nomenclature related to bone histomorphometry dates back to the 1980s, when the first report on this topic was published, to provide standardization and to make comparable results from different studies [12]. Updated standard nomenclature, symbols, and units for bone histomorphometry can be found in the review of Dempster et al. who in 2013 published an update of the Report of the American Society for Bone and Mineral Research (ASBMR) Histomorphometry Nomenclature Committee [13]. In the histomorphometric analysis of bone-implanted medical devices, the useful parameters mostly used are reported in Table 1. The choice of the histomorphometric parameters to be measured is based on the biological and mechanical characteristics of materials implanted in bone and addresses the need to highlight if the purpose for which it has been tested, usually the evaluation of osseointegration degree and osteoconductive/inductive properties, has been reached. As for histological evaluation, the first step in approaching bone histomorphometry for bone-implanted medical devices is the definition of a region of interest (ROI), performing the measures, usually comprising implant site and/or peri-implant bone, and defining a fixed distance from the defect created for the insertion of the material. An example of the identification of the frame of measure is reported in Figure 5(a), representing a hydroxyapatite-coated titanium screw implanted in sheep vertebral pedicle [8–10]. The red frame indicates the ROI within which measures are performed and includes the implant and the peri-implant bone. Sometimes, different ROIs can be taken into account, to perform measures progressively moving away from the implant, as reported in Figure 5(b), in which concentric circles have been depicted around a circular titanium implant, defining the same number of ROI to quantify the bone growth around the material in the iliac crest of a sheep model [8–10]. Once the ROI is defined, it is fixed for all the replicates measured, discarding any samples in which the implant is not in place or that have not been cut according to the correct cutting plane, in order to minimize bias in the measurements. The quantification of area and lengths of bone and materials is at the base of many other complex evaluations to assess key properties of investigated materials. The performance of such measures requires the use of sophisticated image analysis software and specific tools, based on binary image processing, which allow automatic and semiautomatic quantifications. In the correct application of these tools, the efficacy of histological staining is to be mandatory, considering that, as shown in Figure 2(b), different features of the bone can be highlighted by brighter or milder staining, which in turn can be distinguished and quantified by the binarization process. Figure 6 shows an example of image binarization in which the implant is colored in red and the bone in green. The software is able to measure automatically the binarized surfaces, by distinguishing the different colors, quantifying both the area and perimeter. These quantifications can be made either on the entire defined ROI or in specific points of the sample, as in the case of the measure of bone ingrowth, performed on the surface between the two coils of a screw and the line that connects the top of the coils. In Figure 5(c), bone ingrowth measure and the related mirror area, in which bone growth is calculated in the space between the turns of a screw and in the exact specular area, are represented, allowing the estimate of osteoinductive/osteoconductive properties of a metallic implant. On the basis of binarization measures, the Affinity Index or Bone-to-Implant Contact (BIC) can be calculated. This is a classical parameter to assess the degree of osseointegration of an implant, measuring all the points of contact between the material and the bone, as shown in Figure 7, in which the surfaces of contact, manually marked, of a titanium implant in sheep tibial epiphyses are evidenced in orange [8–10]. The analysis software is able to calculate and sum the lengths of each line. One of the major difficulties of many of these evaluations is that manually performed adjustments (for example, after binarization to avoid errors related to a specificity) or measures (as for BIC marks) are necessary, requiring well-trained operators to perform such measures which must be in any case conducted in a double-blind manner.

**Figure 8:**
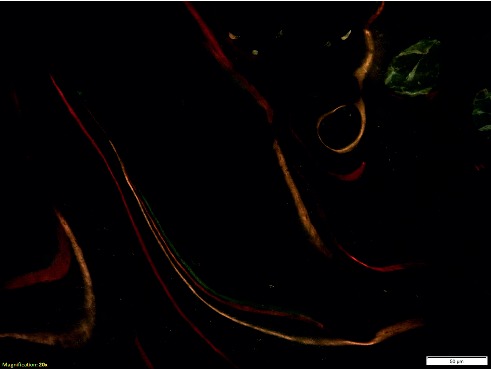
Fluorescence image of rat femur after overtime fluorochrome injections. Along with the profile of bone trabeculae, tetracyclines, xylenol orange, calcein blue, and alizarin red labeling are appreciable (magnification 20x).

**Figure 9:**
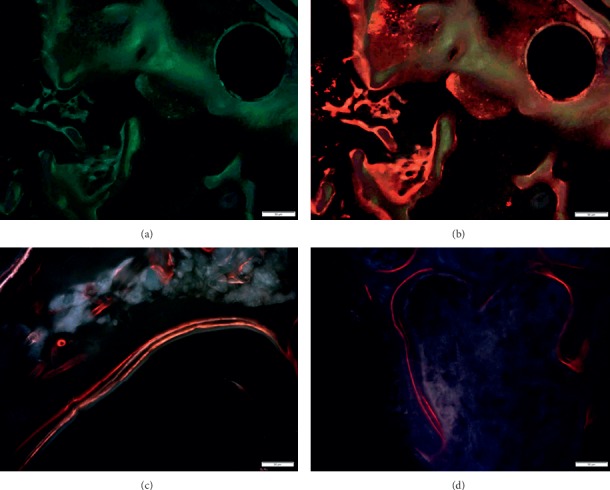
Fluorescence image of alizarin red labeling of rat femur at the interface with implanted material. The double labeling of bone trabeculae is well appreciable (magnification 20x).

High-temperature MMA embedding also allows the histomorphometric evaluation of bone dynamics through the visualization of fluorescent markers. In our laboratory, we employ different fluorochromes types (tetracyclines, xylenol orange, calcein blue, alizarin red) that are administered *in vivo* at scheduled experimental times and are incorporated into the bone matrix during the new bone formation process. Figure 8 is representative of the acquisition in fluorescence of an area of rat femur after the administration of different fluorochromes over time. Bone trabeculae appear labeled progressively in different colors, marking the different steps of bone remodeling. Microscopic observation at appropriate wavelengths for fluorescence emission allows the evaluation of the mineralization status and the time intervals between remodeling processes [14]. Usually, in the presence of an implant, the evaluated ROI is at the interface between the bone and the implant to assess whether the presence of materials has stimulated bone regeneration. Tetracyclines are surely the most used fluorochrome for dynamic evaluation. Its incorporation at the interface of bone and osteoid is seen at a fluorescence microscope as a green line, which becomes double if more than one administration is performed. In Figure 9, alizarin red labeling of rat femoral bone is represented in red color. In the images, the double labeling of bone trabeculae is well evidenced. The length of fluorescent lines and the distance between each other are measurable parameters which can be used to evaluate bone turnover [11, 15].

**Figure 10:**
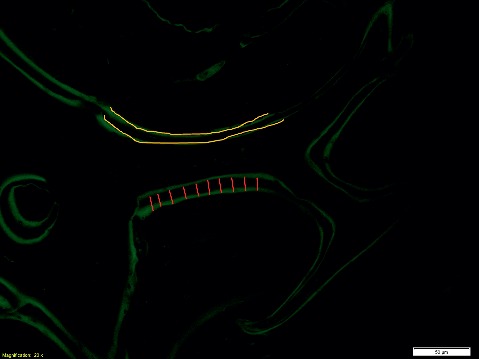
Fluorescence image of tetracycline labeling in sheep tibia, representative of histomorphometrical measure for MAR and BFR assessment. The red lines indicate the measure of the distance between the two bands of the same trabecula, while the yellow lines indicate the length of the fluorescent bands (magnification 20x).

MAR (Mineral Apposition Rate) is the rate at which OBs are making a matrix, which calcifies at a constant rate and incorporates the labels. Thus, it measures the average activity of the OBs in the section. BFR (Bone Formation Rate) takes into account how much of the bone surface is actively mineralizing, which depends on the number of active OBs. It multiplies the average work of each OB by the fraction of bone surface with active OBs. Both of these parameters require manually performed measures of fluorescent bands. In particular, the measure of the distance between the two bands of the same trabecula (red lines), whose average value is combined with the number of days elapsed between the two administrations, the length of the fluorescent bands (yellows lines), and the perimeter of the entire trabecula are shown in Figure 10.

### 3.3. Biomechanical Findings

Biomechanical tests are a fundamental tool in assessing the outcome of biomaterials/device implanted in the bone. Among the evaluation of the osteointegrative process in terms of the new bone formation and bone-to-implant contact, the evaluation of the mechanical competence provides information about the status of the newly formed bone and its ability to support the load. As the final goal is to obtain the formation of bone tissue as similar as possible to the healthy native one, it is clear that mechanical tests are an indispensable evaluation of tissue regeneration. Many of these tests are destructive and need to be performed on fresh or frozen tissue, avoiding the possibility of using the same samples for histological analysis, requiring de facto the use of a huge number of animals. PMMA embedding allows us to perform on the same samples both histology and some mechanical tests, as microhardness and nanoindentation, taking into account, in the final evaluation, that the resin embedding increases microhardness by 30 to 40% [16]. For both techniques, a procedure similar to those applied in the preparation of histological slides is followed, taking care in particular of the polishing step, which needs to be particularly accurate to avoid the presence of surface scratches which can affect the test. Microhardness test is a method for measuring the hardness of a material on a microscopic scale. It is used to provide necessary data when measuring individual microstructures within a larger matrix, or testing very thin foil-like materials, or when determining the hardness gradient of a specimen along a cross section. Samples must fit in the sample stage and be perpendicular to the indenter tip. The microhardness tester needs to be isolated from vibrations. Microhardness analysis, based on the measure of the resistance of the bone to the penetration of a small diamond pyramid, yielded an accurate and reproducible measure of the mineralization degree. The degree of mineralization of bone (DMB) not only influences the mechanical resistance of bone [17] but also partly determines the bone mineral density [18]. Compared to the microhardness test, the nanoindentation test involves smaller loads and thus allows us to investigate a smaller portion of the bone tissue, down to the size of single trabeculae. Typical mechanical parameters obtained from nanoindentation tests are the reduced elastic modulus (*E*_R_) and the indentation contact hardness (*H*_IT_). Noteworthy, elastic modulus and hardness have been found to be well correlated to the degree of mineralization of the tissue [19–21].

## 4. Discussion

The growth of the medical device industry provides numerous novel medical devices, surfaces, biomaterials, scaffolds, and technologies to be used for orthopedic applications. The awareness of the regulatory environment and the need for testing safety, feasibility, and efficacy before clinical use is fundamental to manage the biological assessment of these materials. In this contest, the preclinical evaluation is mandatory and requires a further basic understanding of materials science and bioengineering to facilitate the interpretation of complex interface reactions between biomaterials, cellular and secretory factors, and tissue responses that modulate success or failure of medical devices. Thus, the histological assessment is the irreplaceable requisite for the evaluation of the safety and efficacy of bone-implanted medical devices.

Since the birth of histology and the setup of the techniques for the conservation, processing, and visualization of tissues, the main goal of the discipline has been to find the best way to analyze biological samples, preserving, as best possible, their structure without losing at the same time the possibility of evaluating the characteristics like protein antigenicity, mechanical properties, etc. This attempt has always been particularly complex for the bone tissue, because of its peculiar mineralized structure, which gives unique characteristics of hardness. The increasingly widespread use of biomaterials in orthopedics as the implementation of surgery for traumatic, degenerative, inflammatory, and oncologic diseases has increased the complexity of histological processing, requiring the adoption of embedding methods suitable for cutting without removing the implant, yet it is still able to allow the biological study of the tissue. Histological processing offers a wide variety of options in terms of embedding techniques, allowing choosing the most suitable in the relationship of the tissue analyzed and the outcome to assess.

Paraffin embedding remains the most popular technique for histological evaluation, for its relative ease and speed of execution, requiring a very limited number of items of equipment. However, bone tissues need to be treated with decalcifying solutions to eliminate the mineralized component of the tissue and allow the cutting phase. This procedure is very thorny, as the exact evaluation of the degree of decalcification achieved by the sample is the discriminating factor between obtaining a sample that can be evaluated or not. An excessive decalcification would lead to the destruction of the bone architecture while a too soft decalcification would leave the sample hard, causing the formation of artifacts in the cutting phase. The small dimensions of embedding mold, which are in accordance with the dimensions of cutting microtome, limit the application of this technique to very small samples or to part of them (e.g., biopsies). The only way to evaluate the response of the bone tissue to a material is to mechanically remove the implant and perform the histological and histomorphometrical evaluations taking into account the shadow left by the removed implant [22, 23]. However, this procedure can be feasible only with bulk materials. In fact, porous materials or materials with specific designs, aimed at allowing the bone ingrowth, cannot be removed without damaging the samples and irreparably compromising the evaluation.

Among the various embedding methods available for biological samples, the high-temperature MMA-based technique remains the only one able to address the need for embedding undecalcified samples of big dimensions without removing implanted biomaterial and allowing a satisfying biological evaluation of the tissue. Although this method requires rather long processing times and expensive and not easy to use the equipment for sample preparation, it is still preferable to other embedding solutions that are limited to very small samples and can require the removal of the implant. Among the various resins available, low-temperature MMA is probably the one with the most similar characteristics to the high-temperature MMA, with the advantage of obtaining thinner sections. However, this inclusion technique shares with high-temperature MMA the need for specific equipment and long processing times, but unlike high-temperature MMA, embedding molds allow the processing only of very small samples. Therefore, with the same equipment and procedures, the inclusion in high-temperature MMA allows working on any type of samples, in terms of both the size and type of implants. Although MMA-embedded sections can undergo a variety of histological and histomorphometric evaluations, immunohistochemical analyses still remain a challenge [24]. In fact, though, unlike other resins like GMA, MMA can be deplasticized to perform immunohistochemical analyses [25], the consensus is that the high temperatures reached during MMA polymerization, inducing the formation of reactive radicals, modify the chemical structure of tissues and simultaneously lead to irreversible loss of protein antigenicity. As a way to overcome this pitfall, low-temperature polymerization (+4° to −30°C) using blue or UV light photons as initiators can be used to preserve enzyme activity and protein antigenicity. However, this procedure is obviously highly unsuitable when samples have been *in vivo* labeled with fluorochromes for histomorphometric evaluations in fluorescence. It is well known that the observation of fluorescent histological samples must be conducted with the minimum light exposure and for a short time in order to avoid the decay of the fluorescence emission itself. It is therefore intuitive that this type of polymerization procedure can easily invalidate fluorescence assessments, when required. This aspect proves to be particularly important considering that, despite the incredible advances of nondestructive imaging methods for samples visualization and analysis, like micro positron emission tomography (PET), single-photon emission computed tomography (SPECT), and computed tomography (CT), histomorphometrical analyses remain, to date, an irreplaceable instrument for the evaluation of structural changes of bone tissue. Nevertheless, histological specimens allow the detection and evaluation of biological phenomena as inflammatory reactions, necrosis or hemorrhage, changes in cell morphology, or presence of cellular infiltrate, which cannot be highlighted by radiographic-based imaging acquisition. In addition, considering that the elevated costs of such equipment for animals imaging (i.e., micro-CT/PET) make them not suitable for any laboratories, the use of static histomorphometry stays necessary for the visualization and the measure of the bone structure and dynamic histomorphometry remains an efficient method to evaluate changes over time of the bone in the absence of radiological monitoring. Therefore, despite some limits, the possibility provided by MMA embedding to combine a comprehensive overview of the biological sample and an accurate histological and histomorphometrical analysis makes this method still the best choice for bone-implanted medical device evaluation. Not less important, the embedding in this type of resin also allows further sophisticated evaluations, such as hardness at macro- and nanolevels analyses [26–28]. These techniques are of particular interest in orthopedics as they evaluate if the presence of a material alters the mechanical properties of the surrounding bone and assesses the hardness of a newly formed bone after the implantation of osteoinductive material, allowing an even more detailed and comprehensive assessment of bone-implanted medical devices. The literature on the use of embedded sections is quite varied and not very wide, and, in some cases, the type of embedding medium is not specified [29–31]. Excluding obviously the inclusion in paraffin, which due to the nature of the inclusion medium and the need for decalcification does not lend itself to such tests, the various hard resins available for embedding might be evaluated for the purpose. However, some technical aspects must be considered; for example, GMA is softer than MMA and less suitable for the polishing steps required before microhardness tests [32]. In other cases, the use of other resins (e.g., Epofix3 cold mounting) has required additional steps before performing mechanical tests, like gold coating [33].

Thus, despite many technological advances, microscopical analyses remain an indispensable part of biomedical materials research and part of patient care. The advantages of this technique find their greatest fulfillment in the field of orthopedic translational research, such as that carried out in institutions that conjugate clinical practice and experimental research. In these contexts, in fact, the close and continuous collaboration between clinic and research implies the availability of clinical samples coming from arthroplasty surgery or prostheses substitution for usury or biological failure or from a tumor. Among the diagnostic aspects, which is not addressed in this paper, the possibility of processing and analyzing this type of samples in full allows a thorough and, wherever possible, systematic study over time to further understand the mechanisms underlying in biological reactions to implants and to evaluate mechanical limits and performance. The information related to the osseointegration degree, the presence of adverse reactions, of what kind and to what extent, greater or lesser bone growth, and the evaluations of the quality of the bone, also in terms of mechanical competence, have a great value for identifying the weaknesses and strengths of medical devices, evaluating them in their final application. This can help the design and development of more and more effective and lasting devices, to minimize and avoid, as much as possible, the need of removal or replacement and consequently further surgery, reducing not only the cost burden for the healthcare system but above all the discomfort for the patients.

## Figures and Tables

**Table 1:** Static and dynamic histomorphometric parameters most frequently adopted for the evaluation of bone-implanted medical device.

Material total area	mm^2^	Biomaterial total area
Material total perimeter	mm	Biomaterial total perimeter
Bone area (B.Ar)	mm^2^	Measurement of the amount of trabecular bone
Total area (T.Ar)	mm^2^	Measurement of the total amount of bone observed
Bone volume/Tissue volume (BV/TV)	%	Measure the percentage of spongy bone including the mineralized bone and the osteoid
Bone perimeter (B.Pm)	mm	Length of the observed bone surface
Bone surface/tissue volume (BS/TV)	mm^2^/mm^3^	Measure the percentage of spongy bone surface
Trabecular thickness (Tb.Th)	*µ*m	Thickness of trabeculae derived from the Parfitt formula
Trabecular number (Tb.N)	mm^−1^	Number of trabeculae per surface unit
Trabecular separation (Tb.Sp)	*µ*m	Measure of the distance between the bone trabeculae
Affinity index or bone-to-implant contact (BIC)	%	Length of the areas of direct osseointegration without fibrous capsule interposition
Bone ingrowth	mm^2^	Bone area between the screw and the line that connects the top of the coils/the total area below the top of the coils
Mirror area	mm^2^	Specular image to bone ingrowth
Cortical thickness (Ct.Th)	*µ*m	Cortical thickness
Mineral apposition rate (MAR)	*µ*m/day	Average velocity at which individual osteoid lines are mineralized
BFR/BS	*µ*m^3^/*µ*m^2^/day	Amount of mineralized bone formed per unit of trabecular bone surface per day
